# Supplementary material for: Constitutive Activation of RpoH and the Addition of L-arabinose Influence Antibiotic Sensitivity of PHL628 E. coli
Source: Antibiotics (Basel). 2024 Feb 1;13(2):143. doi: 10.3390/antibiotics13020143 (PMC10886279; doi:10.3390/antibiotics13020143)
Supplement: Supplementary file 1 [file antibiotics-13-00143-s001.zip › antibiotics-2836100-supplementary.pdf]

Supplementary Materials

Constitutive activation of RpoH and the addition of L-arabinose influence antibiotic sensitivity of PHL628 *E. coli*

Jenna K. Frizzell, Ryan L. Taylor, Lisa M. Ryno*

* To whom correspondence should be addressed:
e-mail: lryno@oberlin.edu
Telephone: 440-775-8238
Facsimile: 440-775-6682

Table of Contents

Page

Figure S1: S3

Figure S2: S4

Figure S3: S5

Figure S4: S6

Figure S5: S7

Figure S6: S8

Figure S7: S8

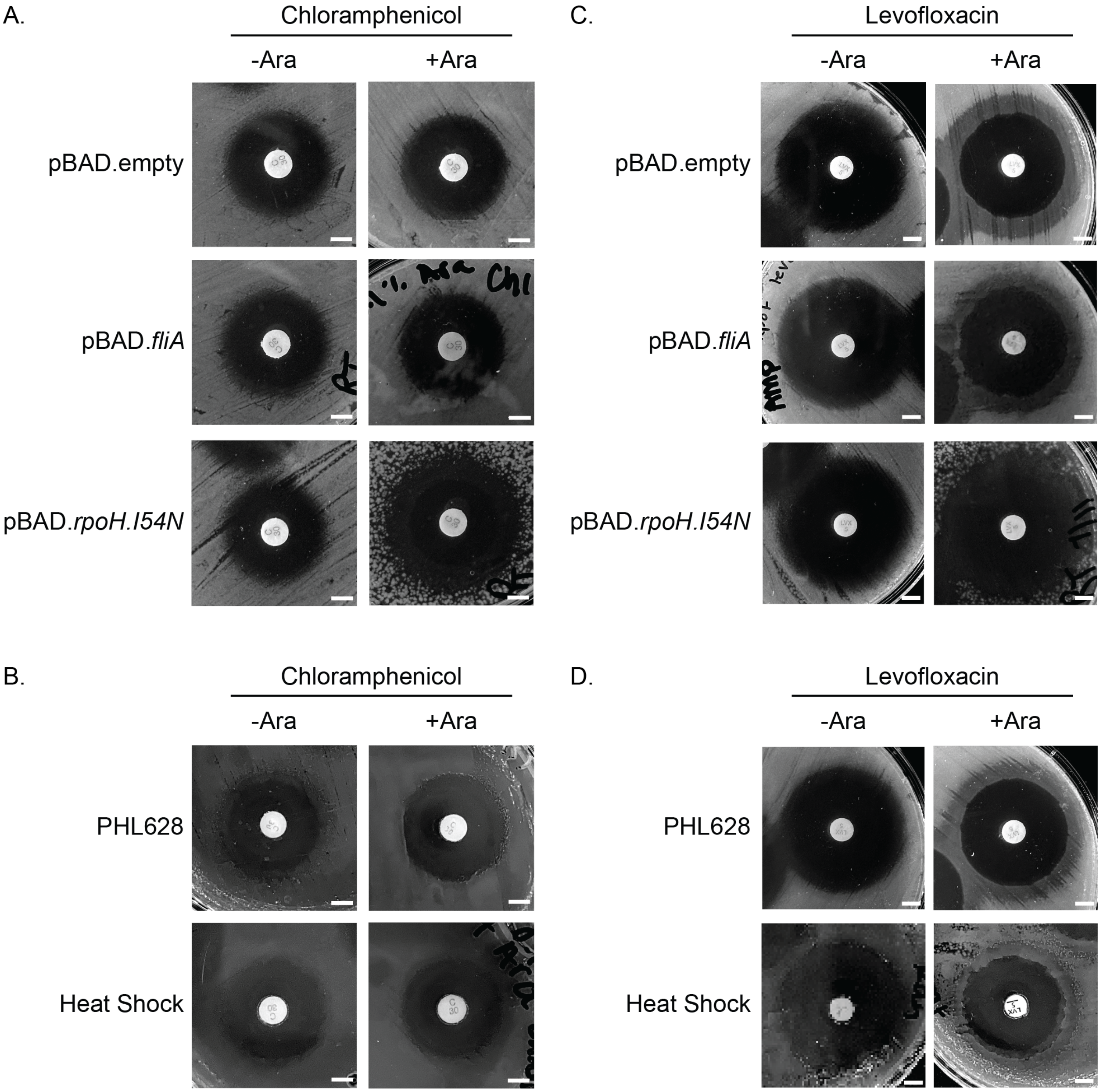

Figure S1. Representative images from disk diffusion assays for 30 μg chloramphenicol treatment of **A.** plasmid-containing cell constructs, or **B.** PHL628 control or heat shocked cells with or without 0.1% (w/w) arabinose. 5 μg levofloxacin treatment of **C.** plasmid-containing or **D.** PHL628 control and heat-shocked cells with or without 0.1% (w/w) arabinose. Scale bar is 0.5 cm.

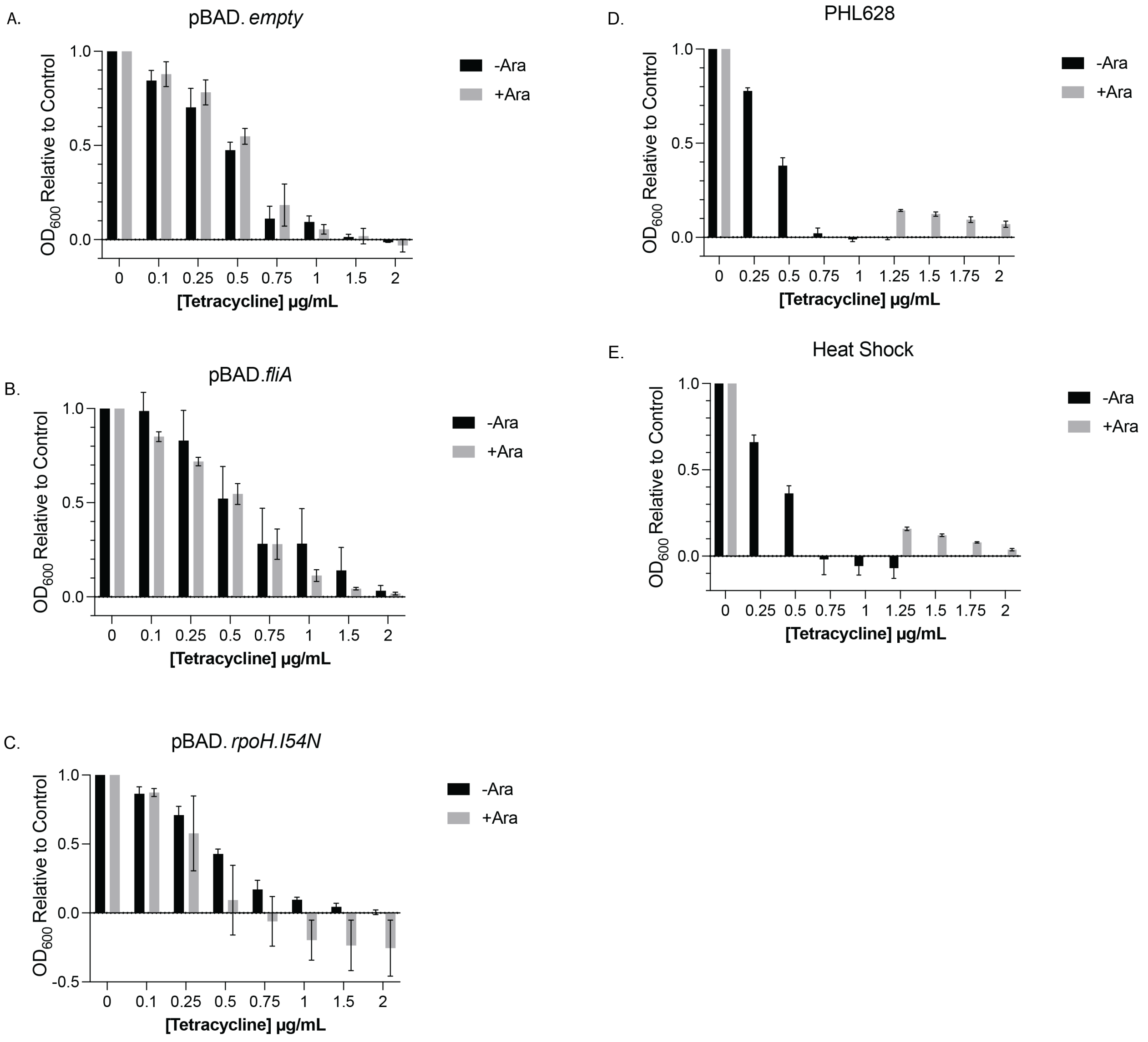

Figure S2. MIC determination experiments for tetracycline for **A.** pBAD.empty, **B.** pBAD.*fliA*, and **C.** pBAD.*rpoH.I54N* constructs. Tetracycline MIC determination for **D.** PHL628 control and **E.** heat shock conditions with or without 0.1% arabinose (N = 3 biological replicates).

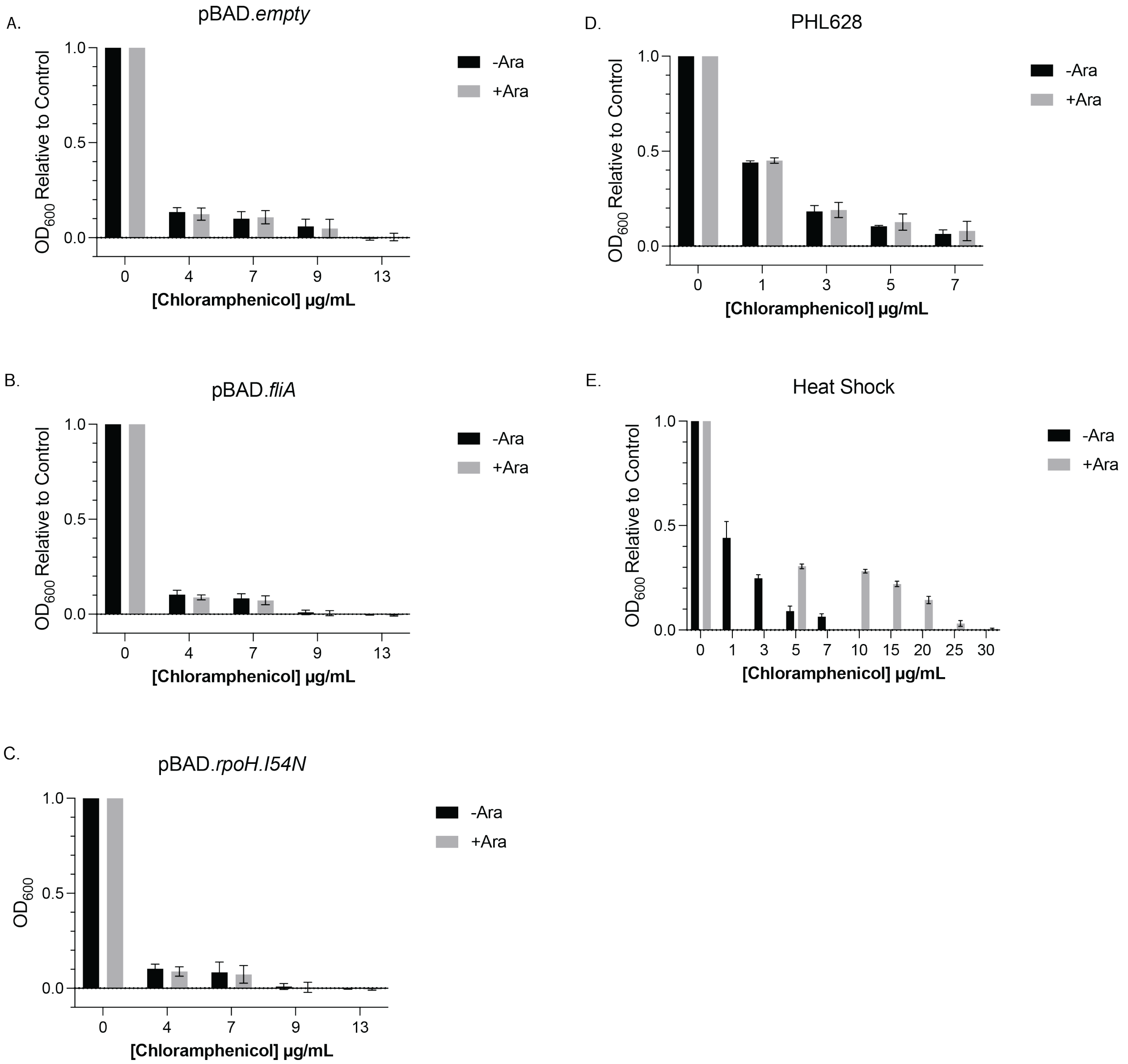

Figure S3. MIC determination experiments for chloramphenicol for **A.** pBAD.*empty,* **B.** pBAD.*fliA*, and **C.** pBAD.*rpoH.I54N* constructs. Tetracycline MIC determination for **D.** PHL628 control and **E.** heat shock conditions with or without 0.1% arabinose (N = 3 biological replicates).

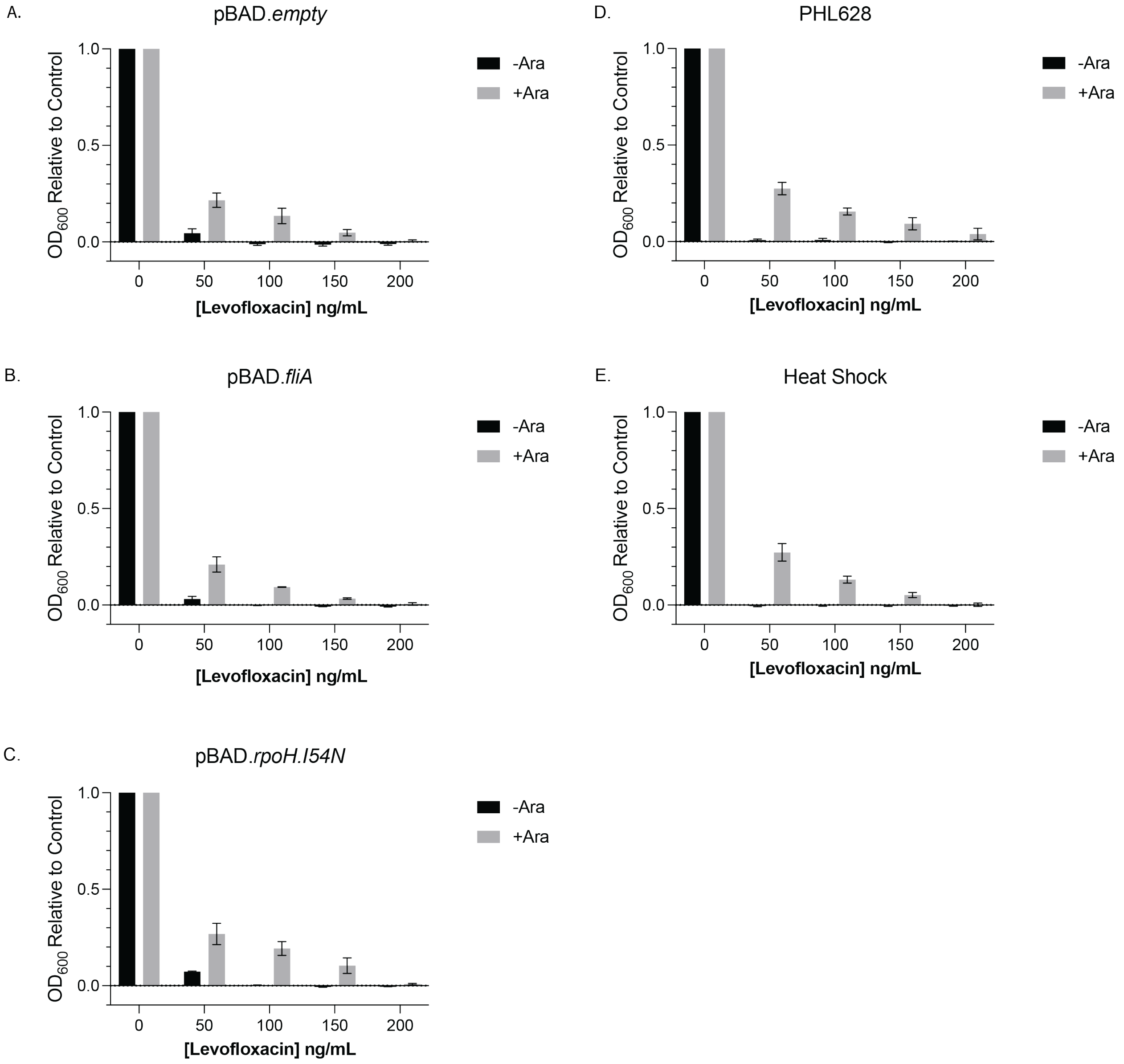

Figure S4. MIC determination experiments for levofloxacin for **A.** pBAD.*empty,* **B.** pBAD.*fliA*, and **C.** pBAD.*rpoH.I54N* constructs. Tetracycline MIC determination for **D.** PHL628 control and **E.** heat shock conditions with or without 0.1% arabinose (N = 3 biological replicates).

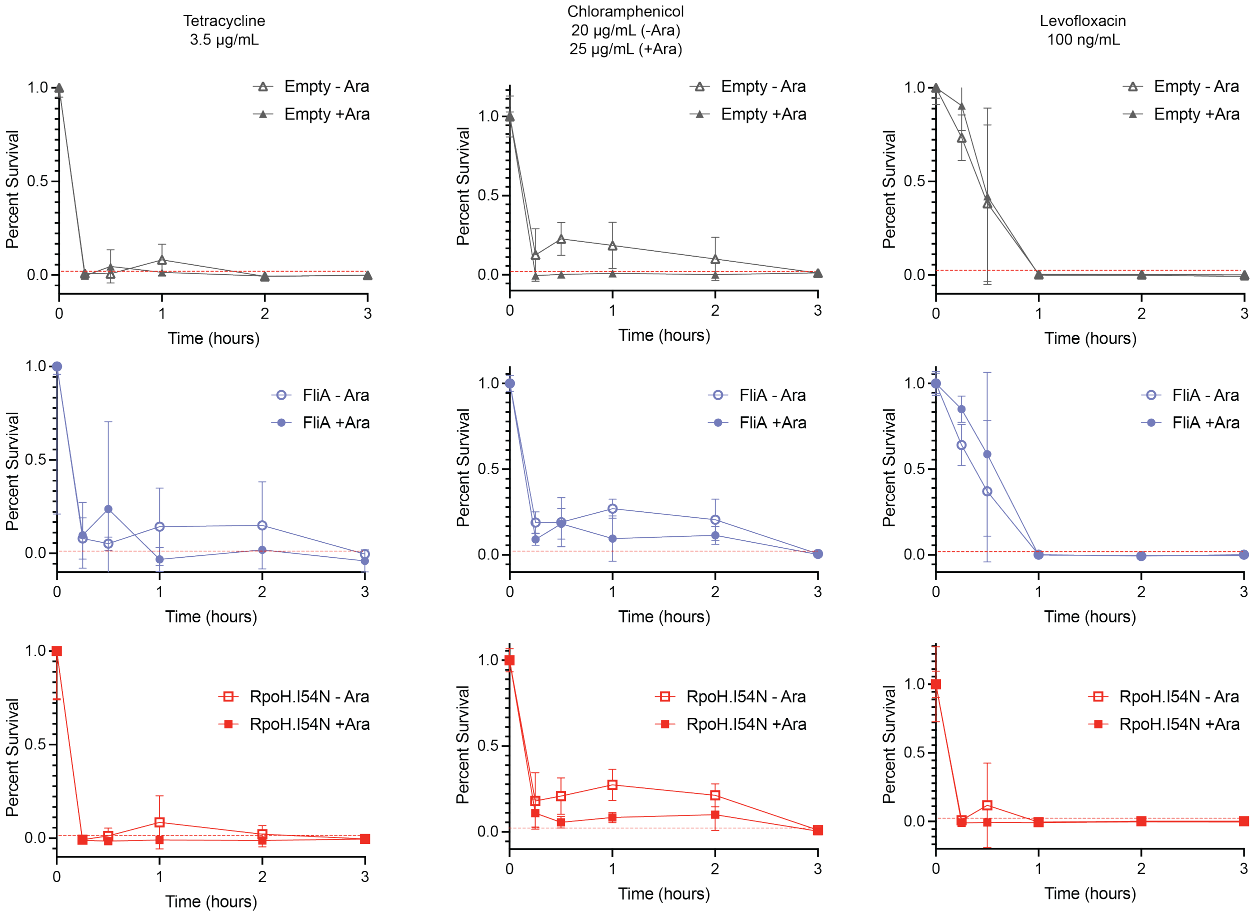

Figure S5. MDK99 Time-Kill Curve Linear Plots for Overexpression Constructs. The acute effect on viability of our transcription factor-overexpressing constructs with **A-C.** 3.5 µg/mL tetracycline +/– arabinose (Ara), **D-F.** 20 µg/mL (-Ara) or 25 µg/mL (+Ara), and **G-I.** 100 ng/mL levofloxacin +/– arabinose (Ara) was confirmed using relative OD_{600} measurements of N ≥ 3 biological replicates. Red dashed lines are at MDK99.

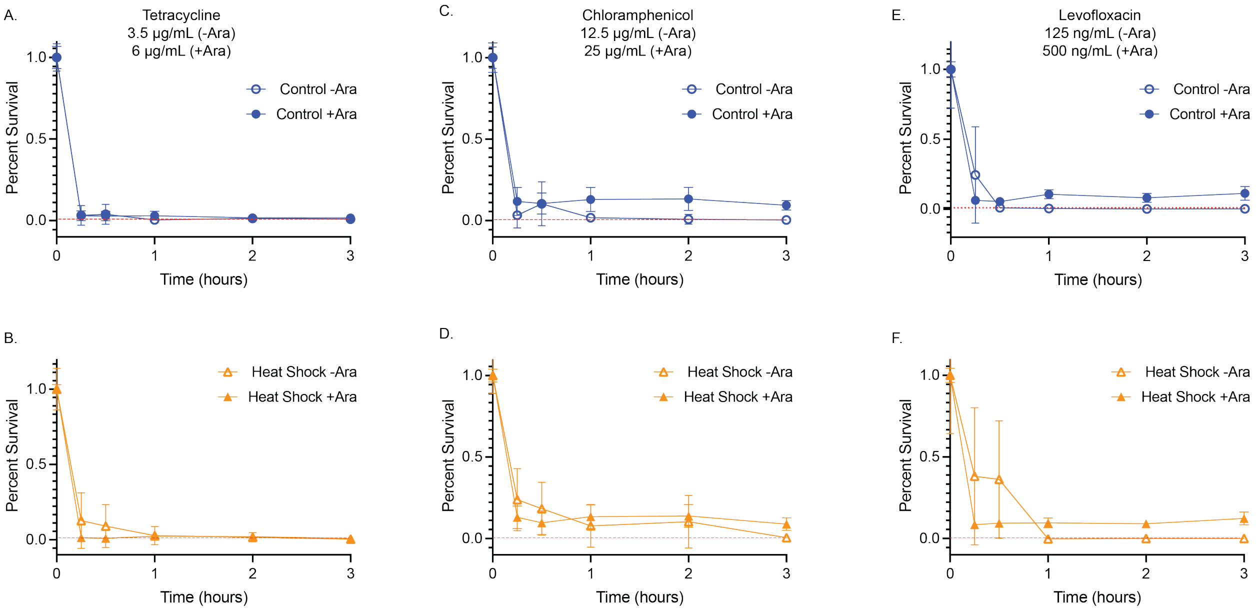

Figure S6. MDK99 Time-Kill Curve Linear Plots for Heat Shock Conditions**.** The acute effect on viability of our control and heat shocked cells with **A-B.** 3.5 µg/mL (–Ara) and 6 µg/mL (+Ara) tetracycline, **C-D.** 12.5 µg/mL (–Ara) or 25 µg/mL (+Ara) chloramphenicol, and **E-F.** 125 ng/mL (–Ara) or 500 ng/mL (+Ara) levofloxacin was confirmed using relative OD_{600} measurements of N ≥ 3 biological replicates. Red dashed lines are at MDK99.

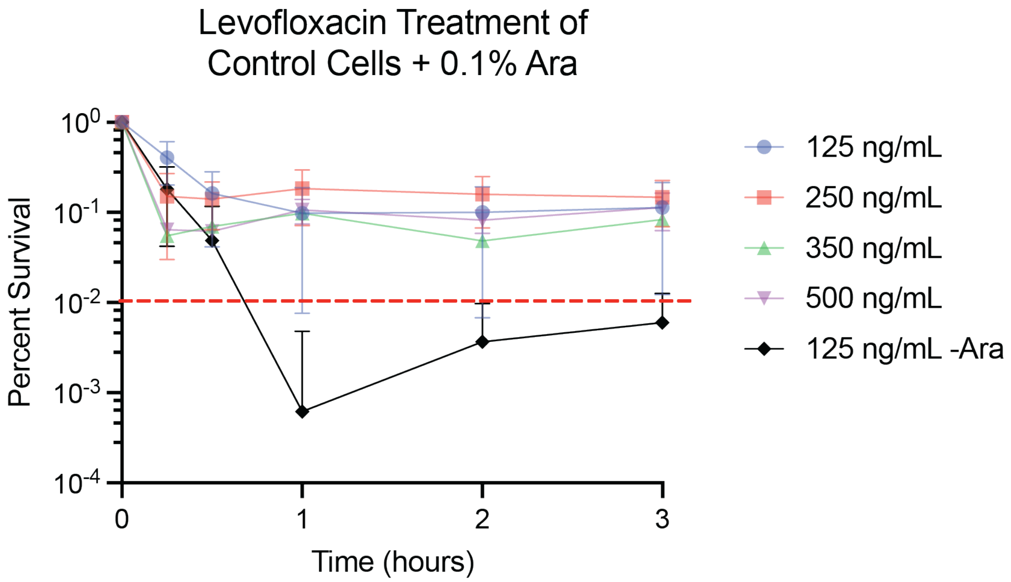

Figure S7. MDK99 Time-Kill Curve Plot for Control Cells. The acute effect on viability of our control cells was assessed for different concentrations of levofloxacin (125 ng/mL – 500 ng/mL) in the presence of 0.1% (w/w) arabinose. Black line is a –Ara control at 125 ng/mL. Dashed red line indicates MDK99 (1% survival). N ≥ 3 biological replicates for all samples except 350 ng/mL levofloxacin (N = 1).
